# A Four-Year Follow-up Study of Renal Epithelioid Angiomyolipoma: A Multi-Center Experience and Literature Review

**DOI:** 10.1038/srep10030

**Published:** 2015-05-05

**Authors:** Jun H. Lei, Liang R. Liu, Qiang Wei, Tu R. Song, Lu Yang, Hai C. Yuan, Yong Jiang, Huan Xu, Sheng H. Xiong, Ping Han

**Affiliations:** 1 Department of Urology, West China Hospital, Sichuan University; 2 Department of pathology, West China Hospital, Sichuan University

## Abstract

In this study, we systematically explored the clinical manifestations, diagnosis, treatment, and prognosis of renal epithelioid angiomyolipoma (EAML) retrospectively by analyzing data of 52 patients diagnosed with EAML at four centers. Our results showed that the onset of EAML was usually inconspicuous, and so no obvious symptoms or signs had occurred in most patients at diagnosis. Its diagnoses always depended on postoperative pathological examination. The immunohistochemical (IHC) results [HMB45 ( + ), cytokeratin (-), and S100 (-)] could be used to differentiate EAML from other malignancies such as renal cell cancer (RCC) and sarcomas. For treatment, surgery resulted in satisfactory short-term prognosis. The long-term prognosis of patients with EAML was poor, particularly when a large size, a high percentage of epithelioid component, tumor thrombus formation, and necrosis were present. In conclusion, EAML is a tumor with malignant potential. Once diagnosed, integrated approaches, including surgery, chemotherapy, and targeted therapy, should be considered; a close follow-up regimen is necessary for cases that met: 1) tumor size >9 cm, 2) tumor thrombus formation in the vein, 3) epithelioid cells >70% or atypia cells >60%, and 4) necrosis.

Renal angiomyolipoma (AML) is one of the most common renal benign tumors, and originates from mesenchymal tissue. Epidemiological studies have revealed that it accounts for approximately 1% of all renal tumors. According to the classification of tumors by the WHO in 2004^1^ there are two types of renal AML: classical and epithelioid. The former is a benign tumor that is composed of different proportions of mature abnormal thick-walled blood vessels, fusiform or epithelioid smooth muscle cells, and adipose tissue. EAML, or monotypic EAML, especially for those with atypia epithelioid cells^2^ has malignant potential, and is mainly composed of a large number of hyperplastic epithelioid cells arranged in sheets; the proportion of mature fat cells tends to be <5%^3^,^4^. EAML can be one part of classical AML, or a single tumor element.

However, most studies assessing malignant EAML are case reports or analyses^5^,^6^; therefore, current understanding of its clinical and pathological characteristics is insufficient. Whether EAML is benign or malignant is controversial, and WHO has not yet commented on whether malignant AML truly exists. Nevertheless, some recent reports raised the concept of malignant EAML because of the histological variability, atypia, and some cases with invasive growth patterns and fatal outcomes.

In the present study, we analyzed the clinical data of 52 patients with atypical EAML who were diagnosed at four centers and performed a literature review to systematically clarify the clinical manifestation, diagnosis, treatment, and prognosis.

## Material and methods

All candidates came from 4 large hospitals in the west of China. Retrospectively, a search with the key words “EAML” (using Chinese) was made in pathology database of each center. The hospitalization and re-examination records of all eligible patients were then reviewed, including clinical features, imaging, type of treatment, and so on. Their paraffin-embedded tissue slides were centralized to the West China Hospital and a central pathological review was conducted by 3 independent pathologists by microscopes (YJ, XH, and SHX). All the paraffin-embedded tissues came from post-operative specimens. The percentage of the epithelioid cells was estimated visually occupying the total tumor areas of the slides and the percentage of the atypia epithelioid cells was estimated visually occupying the total number of epithelioid cells^7^. EAML was defined as polygonal cells with clear to eosinophilic cytoplasm and round to oval nuclei^8^. Atypical EAML was defined as the epithelioid cells with abundant cytoplasm, vesicular nuclei, prominent nucleoli, and nuclear size that exceeds 2 times the size of adjacent nuclei. The degree of nuclear atypia was graded to low, moderate and severe referring to the definition of Fadi *et al.*^7^. Any disagreements were solved through majority voting within the pathological review group. The Follow-up data were achieved by making phone calls or in out-patient department of urology.

Finally, 52 atypical EAML patients were retrieved: the West China Hospital(n = 26), the Sichuan Provincial Cancer Hospital(n = 12), the First Hospital of Guangxi Medical University (n = 10), and the First Affiliated Hospital of Chengdu Medical College(n = 4). 30 patients were male and 22 were female, with a mean age of 39.2 (21–61) years. Twenty-eight tumors involved the left kidney and 24 involved the right kidney. The study was approved by the Ethics Committee of West China Hospital and the methods were carried out in accordance with the approved guidelines. Oral informed consent was obtained for reviewing their pathological specimens when made phone calls or re-examination with each patient. Then, we extracted and analyzed data from the patients’ medical records, postoperative pathological results, and re-examined radiology results [chest X-ray, abdominal computed tomography (CT), and ultrasound] from the inpatient database. Patient survival status was determined by making phone calls. The “Progressors” EAML were defined as the existence of progression, including local recurrence, distal metastasis, and death due to disease.

SPSS 13.0 was used for statistics. Discrete variables and continuous variables were compared with the Chi-Square and *t* test, respectively.

## Results

Of the 52 patients enrolled, we were unable to determine the survival status of 9: five did not provide or provided unavailable phone numbers, and four didn't want to cooperate. Finally, we obtained the complete clinical data of 43 patients (83%), with a mean follow-up duration of 44.3 (4–71) months. The clinical data of follow-up patients in details were summarized in Table 1. The compare of pathologic features between progressors and non-progressors EAML was shown at Table 2.

When the surgical treatment of the 43 cases in the present study are summarized, 12 underwent radical nephrectomy, 30 used nephron-sparing surgery, and one underwent radical nephrectomy with the removal of the tumor thrombus in postcava. After follow-up of approximate 4 years, one patient underwent radical nephrectomy and one underwent nephron-sparing surgery suffered metastasis or recurrence; all other patients, including one with liver metastasis at baseline were alive. AML specimens from 43 patients were stratified into 3 groups based on the percentage of epithelioid/atypia epithelioid cells. Among the six cases with >90% epithelioid cells, three showed obvious focal necrosis and hemorrhage. The tumor cells showed different degrees of polymorphy, with irregular shape that differed from the epithelioid or classical AML. Mitotic figures were readily identified and karyolobism could be seen.

**Figure 1:**
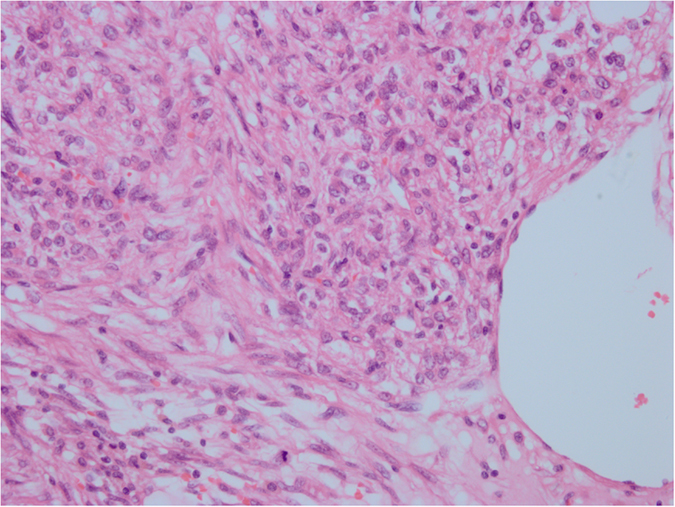
HE staining. The ranges of vision are filled with abundant epithelioid cells, with red cytoplasm and large nuclei; nucleoli were visible, with a certain degree of pleomorphism and mitotic figures. Original magnification ×400.

**Figure 2:**
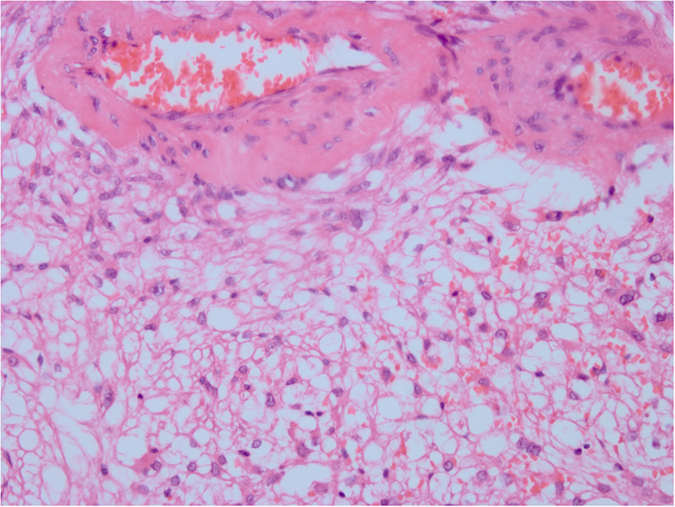
HE staining. The epithelioid cells are diffuse, surrounded by a great deal of fatty cells. Original magnification ×400.

**Figure 3:**
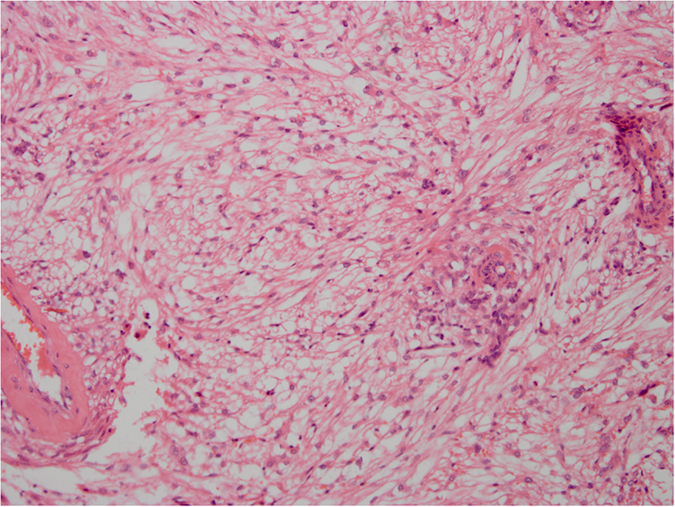
HE staining. The epithelioid cells are not distributed uniformly. Atypical epithelioid cells with abundant cytoplasm, vesicular nuclei, prominent nucleoli, and large nuclear size can be seen. Original magnification ×200.

Among these three, two had renal hilum and para-aortic lymph node metastasis at diagnosis. During the follow-up, one of the two patients with lymph node metastasis was diagnosed with hepatic and lung metastasis 12 months postoperatively, and died 17 months later (Fig. 1); one patient with renal hilus node metastasis at diagnosis suffered local recurrence 10 months postoperatively and died from serious cancer cachexia 4 months later (Fig. 2); the third case (Fig. 3) and all the others did not suffer any signs of progression and live well.

## Discussion

Although some researchers still consider renal AML to be a hamartoma, a tumor-like malformation formed by aberrantly assembled normal renal tissues, many studies have unequivocally demonstrated that it is a tumor. Paradis *et al.* 9 and Green *et al.*^10^ revealed its clonal origination by revealing that chromosome X was inactivated randomly. Kattar *et al.*^11^ reported gene deficiencies in chromosome band 16p13 and clonal chromosomal aberrations in other specified locations. Therefore, the latest WHO classification of diseases in 2004 announced that the renal AML was tumor. It also defined EAML as a potentially malignant mesenchymal neoplasm characterized by the proliferation of predominantly epithelioid cells. However, no previous studies have defined clear lower limit of epithelioid cells’ percentage of EAML, which could be called a “real” EAML. Some used the phrase “predominantly” or “mostly”^12^,^13^,^14^,^15^,^16^, and others used “pure”^17^. In the present study, the cases included were stratified into 3 groups based on proportion of epithelioid/atypical epithelioid cells that were present. Herein, we attempted to 1)systematically clarify the clinical manifestation, diagnosis, treatment, and prognosis; 2)unfolded the potiential relationship between proportion of epithelioid/atypical epithelioid cells and progression of EAML, as discussed below.

### Clinical manifestation

Similar to patients with classical AML, patients with EAML show an insidious onset, and tumors are often discovered in imaging results from physical examinations. Dickinson *et al.*^18^ discovered that 82%–94% of patients with lesions over 4 cm complained of clinical symptoms, and 50%–60% had concomitant bleeding. Of the 43 cases in this study, 8 visited physicians because of significant symptoms or signs: 6 with backache, and 2 with hematuria. All of these four tumors were larger than 5 cm. One hematuria case and one backache case were proved to be malignant. These data suggest that the larger the lesion is, the greater the probability of clinical symptoms. Based on our intraoperative findings and postoperative pathological reports, almost all EAMLs had an abundant blood supply. The blood vessels supplying the tumors often communicated with the vessels supplying the adrenal gland, urinary duct, or diaphragm, and were usually tortuous with a missing internal elastic membrane. This phenomenon might explain why most cases had different levels of localized hemorrhagic necrosis, which is consistent with the results of Yamakado *et al.*^19^.

### Diagnosis

Making a preoperative diagnosis for EAML is challenging for several reasons; therefore, it is often misdiagnosed as RCC. This is in part because EAML also has an insidious onset, and no specific clinical manifestation. In addition, imaging examination techniques such as CT and MRI are sensitive to adipose tissue, and the amount of adipose tissue in EAML is <5%^3^,^4^. Therefore, its diagnosis often depends on postoperative pathological examinations when epithelioid cells are detected. Nevertheless, the application of Doppler ultrasound or CT can pick up incidental tumours in asymptomatic patients. Previous studies^20^,^21^ demonstrated that EAML was positive for melanoma cell markers (such as HMB45) and smooth muscle cell markers, but negative for epithelial cell markers. Pea *et al.*^18^ and L’Hostis *et al.*^22^ reported four cases of EAML: three were diagnosed with RCC and one with classical AML preoperatively. However, all cases were diagnosed as EAML based on postoperative HMB45 expression and pathological results.

Under a microscope, epithelioid cells from the 43 cases in the present study were irregular polygon shaped, with enlarged pleomorphic nuclei in the mitotic phase and cytoplasm enriched with eosinophil granules. In addition, IHC results revealed that all 43 cases were HMB45-positive. However, other malignancies such as RCC and sarcomas do not express HMB-45. Ooi *et al.*^23^ demonstrated that because Ki-67 is involved in cell proliferation, it could serve as an antigen to indicate the cell proliferation status during the diagnosis of EAML. However, the present study revealed that Ki-67 had a low diagnostic sensitivity because only 10 of 43 cases were positive for Ki-67, and approximately 2–10% cells were stained. Other markers such as cytokeratin and S100 were usually negative. So, the differential expression of HMB-45, cytokeratin and S100 can be used to differentiate EAML from RCC and metastatic melanoma^5^.

### Treatment

To date, no approaches are more effective than surgery for EAML; however, surgery is commonly insufficient. Many previous studies^20^,^24^,^25^,^26^,^27^,^28^,^34^ have reported that local recurrence or distant metastasis generally occur 1.5–9 years postoperatively. Zomboni *et al.*^29^ reported that EAML belonged to a family of perivascular epithelioid cell tumors (PEComa), similar to lymphagioleiomyomas and clear cell “sugar” tumors of the lung and pancreas. Therefore, EAML might be sensitive to chemotherapy, similar to the other two tumor types. Cibas *et al.*^30^ and Park *et al.*^12^ used doxorubicin, cyclophosphamide, and cisplatin to treat EAML, and they found that the tumors were responsive. However, long-term follow-up observation was needed to confirm their effects. Heidi *et al.*^31^ studied 15 cases of PEComa, and found that the mammal rapamycin (mTOR) cascade was always activated, which is related to tumor growth and development. This suggests that patients might benefit from mTOR inhibitors and that further studies should be performed urgently.

Based on the current cohort, we believed that nephron-sparing nephrectomy was safe for patients without lymph node metastasis and tumor thrombus formation; nevertheless, long-term survival requires the support of long-term follow-up data. Multiple therapies, including surgery, chemotherapy, and molecular-targeted drugs, might be beneficial for long-term patient survival.

### Prognosis

Previous data revealed that patients with EAML had poor prognosis. After Martignoni *et al.*^31^ first reported fatal cases of EAML in 1994, many case reports^20^,^24^,^25^,^26^,^27^,^28^,^34^demonstrated that EAML had malignant potential and might cause patient mortality. Distant metastases were found in different organs 1.5–9 years postoperatively, as confirmed by biopsy or autopsy, and most patients died within 1 year of metastasis^24^,^25^,^26^,^27^,^28^. Tsai *et al.*^33^ reported that at least 30% of EAML patients would progress to typical malignant EAML. Vaema *et al.*^34^ performed a systematic review of 10 previously published case studies and, regardless of potential publication bias, they found that most patients died from tumor progression, and many included liver and lung metastasis.

Nevertheless, the biological behavior of EAML remains controversial. According to the WHO definition in 2004, EAML are tumors with malignant potential. To predict patient prognosis earlier, some recent studies introduced prediction models for malignant EAML after studying a large number of samples. Fadi *et al.*^7^ first defined atypical epithelioid cells (AECs). They then summarized 40 cases of atypical EAML, and presented a prediction model for malignant EAML as follows: 1) percentage of AEC ≥70% EC, 2) ≥2 mitotic figures per 10 high power fields (HPF), 3) atypical mitotic figures, and 4) necrosis. The model focused on the pathological features of the AEC nuclei and 78% of EAML cases who finally occurred recurrence or metastasis were involved. Therefore, the model had a high reference value. Nese *et al.*^17^ summarized 41 cases of “pure” EAML (epithelioid cells accounted for almost 100% of the tumor). They reported that the following five characteristics were related to tumor progression: 1) tuberous sclerosis complications, 2) tumor size >7 cm, 3) involvement of the perirenal and/or renal vein, 4) a carcinoma-like growth pattern, and 5) necrosis. After their follow-up, tumor progression was seen all in tumors that fulfilled more than four of these five characteristics. The model by Nese *et al.*, which places emphasis on general features, seems to have more accurate predictive power than the model by Fadi *et al.* However, it must be considered that Nese’s model was based on “pure” EAML. Therefore, the percentage of epithelioid cells might be associated with tumor progression and a higher probability that all five characteristics would occur. Faraji *et al.*^35^ also found that tumors with cytological atypia and tumor necrosis showed an unfavorable outcome.

Our study demonstrated that, after a follow-up of 44.3 months, only 2 patients with node metastases and embolus formation suffered recurrence or metastasis and died finally. The two fatal cases were consistent with the results of previous fatal cases^32^ in which liver, lung, or bone metastases occurred frequently. The remaining 41 patients, including one patient with liver or bowel metastasis before the surgery, survived. When the 3 progressors were compared with non-progressors (Table 2), it was obvious that the fatal case had a significantly higher percentage of epithelioid cells, percentage of atypia cells, and Max diameter. This may enhance the concept that a higher percentage of epithelioid cells facilitates tumor progression. The multi-center study performed by Nese *et al.*^17^, which included 41 “pure” EAML cases, revealed recurrence or metastasis in 50% of patients, and 30% eventually died from their disease. One explanation for the low percentage of progression in this 43 cohort might be the low percentage of epithelial cells (mean: 43.8 ± 22.2). Another explanation could be the limitation of the present study of a relatively short follow-up period, because a previous case^34^ did not exhibit distant metastasis until after 9 years of follow-up. In the present study, the three progressors cases met the following characteristics concerning to general and pathological features: 1) tumor size >9 cm, 2) tumor thrombus formation in the vein, 3) epithelioid cells >70% or atypia cells >60%, and 4) necrosis. A lesion that met 3 or more of the above features predicted increased risk of malignant behavior and should clinically be closely followed.

### Limitations

Given the rare nature of the disease, prospective research design was difficult to conduct. This was the inherent limitation of the study. For this reason, we included patients’ cohort from different hospitals retrospectively. Also, the proper option of surgical method (eg. radical or nephron-sparing nephrectomy) was not available. We couldn’t establish uniform criterion for surgical extirpation; the histopathology of these lesions was mostly determined postoperatively. Therefore, more convincing data from prospective, multicenter, cohort study may reveal the effect of surgical method to prognosis.

## Conclusions

EAML is a tumor with potential malignancy. Once diagnosed, particularly when a tumor met the above characteristics like the three malignant cases, active treatment, including radical nephrectomy, chemotherapy, and molecular-targeted drugs should be considered and patients should be closely followed.

## Author Contributions

JHL and LRL wrote the first edition of the paper. TRS, LY, and HCY obtained patient consent and collected data. YJ, XH, and SHX analyzed the pathological results. PH and QW commented in detail on the drafts and approved the final version.

## Additional Information

**How to cite this article**: Lei, J. H. *et al*. A Four-Year Follow-up Study of Renal Epithelioid Angiomyolipoma: A Multi-Center Experience and Literature Review. *Sci. Rep.* 5, 10030; doi: 10.1038/srep10030 (2015).

## Figures and Tables

**Table 1:** Clinical data of follow-up patients diagnosed with EAML (n = 43).

**Baseline data**	
* Gender*	male/female=25/18
* Mean age*/years	38.4 (24–76)
* Max diameter*	≤4cm: n=29; 4–7 cm: n=4; 7–10 cm: n=7; ≥10 cm: n=3
* percentage of epithelioid cells*	>90%: n=6; 70–90%: n=9; 5%~70%: n=28. mean: 43.8±22.2
* percentage of atypia cells*	>90%: n=2; 70–90%: n=5 ; 5%~70%: n=36. mean: 28.4±23.1
* Degree of atypia*	severe: n=4; moderate: n=10; low: n=29
* Necrosis*	prominent: 21; focal: 16; little: 6
* Associated symptoms/signs*	backaches: n=6; hematuria: n=2; without: n=35
	
*Involvement range*	
* *confined to kidney(n=35)	Right: n=19 left: n=16
* *beyond kidney (n=6)	perinephric fat: n=3; renal vein: n=2; infraphrenic postcava: n=1
* *distant metastasis(n=2)	liver: n=1; bowel: n=1
* tumor embolus*	(+) for 3 cases (1 in infraphrenic postcava; 2 in renal vein)
* Lymph nodes metastasis*	renal hilus node: n=1; renal hilus and para-aortic nodes: n=1
* Distant metastasis*	liver: n=1; bowel: n=1
* Surgical method*	radical nephrectomy: n=12; nephron-sparing surgery: n=30;radical nephrectomy + removal of tumor thrombus in postcava: n=1
	
**IHC stain**	
* *HMB-45$	39 (+); 4(focal +)
* *Ki-67&	10 (+, 2–10%); 28 (−); 5 (unclear)
* *S100⊠	40 (−); 3 (unclear)
	
**Follow-up data**	
* Follow up duration/month*	44.3 (4−71)
* survival status*	alive : n=41; dead: n=2
* Recurrence*	One patient with renal hilus node metastasis at baseline suffered local *recurr*ence 10 months postoperatively and died from serious cancer cachexia 4 months later
* Metastasis*	One patient suffered metastasis to the liver and lung 12 mons postoperatively and died 17 months after metastasis

^$^HMB-45 is a monoclonal antibody that reacts against an antigen present in melanocytic tumors such as melanomas, and means Human Melanoma Black.

^&^KI-67 is a nuclear protein that is associated with and may be necessary for cellular proliferation.

^⊠^S100 is a kind of melanoma marker.

**Table 2:** The compare of pathologic features between Non-progressors and Progressors.

**Mean**	**Non-progressors (n = 40)**	**Progressors (n = 3)**	**P**⊠
*Age/years*	43.4	45	0.853
Ratio of male/female	1.4(23/17)	2(2/1)	1.0
*Max diameter/cm*	3.3	10	<0.001
*Percentage of epithelioid cells*	40.9	83.3	0.001
*Percentage of atypia cells*	24.8	76.7	<0.001

^⊠^Chi-Square test was used for Ratio of male/female; *t* test were used for the others.

## References

[b1] Eble J. N. *et al.* World Health Organization classification of tumours. Pathology and genetics of tumours of the urinary system and male genital organs [943–945] IARc Press: Lyon, 2004.

[b2] Lopez-Beltran A. *et al.* 2004 WHO Classificationof the Renal Tumors. Eur. Urol. 49, 798–805 (2004). 16442207 10.1016/j.eururo.2005.11.035

[b3] Halpenny D., Snow A. & McNeill G. The radiological diagnosis and treatment of renal angiomyolipomadcurrent status. Clin. Radiol. 65, 99–108 (2010). 20103431 10.1016/j.crad.2009.09.014

[b4] Kuo-How H. *et al.* Malignant Epithelioid Angiomyolipoma of the Kidney. J. Formos. Med. Assoc. 106, 51–54(2007). 17493897 10.1016/s0929-6646(09)60353-3

[b5] Acikalin M. F. *et al.* Epithelioid angiomyolipoma of the kidney. Int. J. Urol. 12, 204–7 (2005). 15733117 10.1111/j.1442-2042.2005.01003.x

[b6] Chia-Chun T. *et al.* Epithelioid angiomyolipoma of the kidney mimicking renal cell carcinoma: a clinicopathologic analysis of cases and literature review. Kaohsiung J. Med. Sci. 25, 133–40 (2009). 19419918 10.1016/S1607-551X(09)70052-X PMC11918126

[b7] Brimo F. *et al.* Renal Epithelioid Angiomyolipoma With Atypia: A Series of 40 Cases With Emphasis on Clinicopathologic Prognostic Indicators of Malignancy. Am J Surg Pathol 34, 715–22 (2010). 20410812 10.1097/PAS.0b013e3181d90370

[b8] Aydin H. *et al.* Renal Angiomyolipoma Clinicopathologic Study of 194 Cases with Emphasis on the Epithelioid Histology and Tuberous Sclerosis Association. Am J. Surg. Pathol. 33, 289–297 (2009). 18852677 10.1097/PAS.0b013e31817ed7a6

[b9] Paradis V. *et al.* Clonal analysis of renal sporadic angiomyolipomas. Hum. Pathol. 29, 1063–67 (1998). 9781642 10.1016/s0046-8177(98)90414-2

[b10] Green A. J., Sepp T. & Yates J. R. Clonality of tuberous sclerosis hamartomas shown by non-random X-chromosome inactivation. Hum. Genet. 97, 240–243 (1996). 8566961 10.1007/BF02265273

[b11] Kattar M. *et al.* Chromosomal analysis of renal angiomyolipoma by comparative genomic hybridization: evidence for clonal origin. Hum. Pathol. 30, 295–99 (1999). 10088548 10.1016/s0046-8177(99)90008-4

[b12] Park H. K. *et al.* Clinical presentation of epithelioid angiomyolipoma. Int. J. Urol. 14, 21–5 (2007). 17199855 10.1111/j.1442-2042.2006.01665.x

[b13] Belanger E. C. *et al.* Epithelioid angiomyolipoma of the kidney mimickingrenal sarcoma. Histopathology 47, 433–5 (2005). 16178901 10.1111/j.1365-2559.2005.02134.x

[b14] Takahashi N. *et al.* Malignant transformation of renal angiomyolipoma. Int. J. Urol. 10, 271–3 (2003). 12694468 10.1046/j.1442-2042.2003.00620.x

[b15] Pea M. *et al.* Apparent renal cell carcinomas in tuberous sclerosis are heterogeneous: the identification of malignant epithelioid angiomyolipoma. Am J. Surg. Pathol. 22, 180–7 (1998). 9500218 10.1097/00000478-199802000-00005

[b16] Martignoni G. *et al.* Carcinoma like monotypic epithelioid angiomyolipoma in patients without evidence of tuberous sclerosis: a clinicopathologic and genetic study. Am J. Surg. Pathol. 22, 663–72 (1998). 9630173 10.1097/00000478-199806000-00003

[b17] Nese N. *et al.* Pure Epithelioid PEComas (So-Called Epithelioid Angiomyolipoma) of the Kidney: A Clinicopathologic Study of 41 Cases: Detailed Assessment of Morphology and Risk Stratification. Am J. Surg. Pathol. 35, 161–76 (2011). 21263237 10.1097/PAS.0b013e318206f2a9

[b18] Dickinson M. *et al.* Renal angiomyolipoma: optimal treatment based on size and symptoms. Clin. Nephrol. 49, 281–6 (1998). 9617489

[b19] Yamakado K. *et al.* Renal angiomyolipoma: relationships between tumor size, aneurysm formation, and rupture. Radiology 225, 78–82 (2002). 12354988 10.1148/radiol.2251011477

[b20] Martignoni G. *et al.* Renal epithelioid oxyphilic neoplasm (REON): a pleomorphic monophasic variant of renal angiomyolipoma. Int. J. Surg. Pathol. 2, 539 (1994).

[b21] Pea M. *et al.* Apparent renal cell carcinomas in tuberous sclerosis are heterogeneous: the identificationof malignant epithelioid angiomyolipoma. Am J. Surg. Pathol. 22, 180–87 (1998). 9500218 10.1097/00000478-199802000-00005

[b22] L’Hostis M. *et al.* Renal angiomyolipoma: a clinicopathologic, immunohistochemical, and follow up study of 46 cases. Am J. Surg. Pathol. 23, 1011–20 (1999). 10478660 10.1097/00000478-199909000-00003

[b23] Ooi S. M., Vivian J. B. & Cohen R. J. The use of the Ki-67 marker in the pathological diagnosis of the epithelioid variant of renal angiomyolipoma. Int. Urol. Nephrol. 41, 559–65 (2009). 18839327 10.1007/s11255-008-9473-1

[b24] Mai K. T., Perkins D. G. & Collins J. P. Epithelioid cell variant of renal angiomyolipoma. Histopathology 28, 277–80 (1996). 8729052 10.1046/j.1365-2559.1996.d01-421.x

[b25] Bjorsson J. *et al.* Tuberous sclerosis-associated renal cell carcinoma, clinical, pathological and genetic features. Am J. Pathol. 149, 1201–8 (1996). 8863669 PMC1865172

[b26] Takumi Y. *et al.* Rapidly progressive malignant epithelioid angiomyolipoma of the kidney. J. Urol. 168, 190–1 (2002). 12050523

[b27] Huang K. H. *et al.* Malignant epithelioid angiomyolipoma of the kidney. J Formos. Med. Assoc. 106, 51–54 (2007). 17493897 10.1016/s0929-6646(09)60353-3

[b28] Hardman J. A. *et al.* Recurrent renalangiomyolipoma associated renal carcinoma in a patient with tuberous sclerosis. Br. J. Urol. 72, 983–4 (1993). 8306177 10.1111/j.1464-410x.1993.tb16321.x

[b29] Zamboni G. *et al.* Clear cell “sugar” tumor of the pancreas. A novel member of the family of lesions characterized by the presence of perivascular epithelioid cells. Am J. Surg. Pathol. 20, 722–30 (1996). 8651352 10.1097/00000478-199606000-00010

[b30] Cibas E. S. *et al.* Malignant epithelioid angiomyolipoma (‘sarcoma ex angiomyolipoma’) of the kidney: a case report and review of the literature. Am J. Surg. Pathol. 25, 121–6 (2001). 11145246 10.1097/00000478-200101000-00014

[b31] Heidi K. *et al.* Activation of the mTOR pathway in sporadic angiomyolipomas and other perivascular epithelioid cell neoplasms. Hum. Pathol. 38, 1361–71 (2007). 17521703 10.1016/j.humpath.2007.01.028 PMC2722219

[b32] Martignoni G. *et al.* Renal epithelioid oxyphilic neoplasm (REON): a pleomorphic monophasic variant of renal angiomyolipoma. Int. J. Surg. Pathol. 2, 539 (1994).

[b33] Tsai C. C. *et al.* Epithelioid angiomyolipoma of the kidney mimicking renal cell carcinoma:a clinicopathologic analysis of cases and literature review. Kaohsiung J. Med. Sci. 25, 133–40 (2009). 19419918 10.1016/S1607-551X(09)70052-X PMC11918126

[b34] Varma S. *et al.* Renal epithelioid angiomyolipoma: a malignant disease. J. Nephrol. 24, 18–22 (2011). 20349413 10.5301/jn.2010.5451

[b35] Faraji H., Nguyen B. N. & Mai K. T. Renal epithelioid angiomyolipoma: a study of six cases and a meta-analytic study. Development of criteria for screening the entity with prognostic significance. Histopathology 55, 525–534 (2009). 19912358 10.1111/j.1365-2559.2009.03420.x

